# Supplementary material for: MetaRibo-Seq measures translation in microbiomes
Source: Nat Commun. 2020 Jun 29;11:3268. doi: 10.1038/s41467-020-17081-z (PMC7324362; doi:10.1038/s41467-020-17081-z)
Supplement: Supplementary file 10 — Supplementary Data 7 [file 41467_2020_17081_MOESM10_ESM.zip › File2/Confidence_High_Taxonomy/699_out.krona.html]

Javascript must be enabled to view this page.

members
magnitude
magnitudeUnassigned
count
unassigned
taxon
rank

699\_out

7

2
superkingdom
7

phylum
1239
7

7
186801
class

7
186802
order

541000
family
7

genus
1263
7

165186
species
7

SRS011134\_contig\_number\_46449SRS011302\_contig\_number\_15271SRS013638\_contig\_number\_17991SRS018313\_contig\_number\_10081SRS022609\_contig\_number\_48024SRS053335\_contig\_number\_2412SRS104197\_contig\_number\_contig-100\_7.3057
